# Prostatic urethra malformation associated with retrograde ejaculation: a case report

**DOI:** 10.1186/s13256-016-1150-x

**Published:** 2016-12-21

**Authors:** Kai Zhao, Jianzhong Zhang, Aiming Xu, Cheng Zhang, Zengjun Wang

**Affiliations:** State Key Laboratory of Reproductive Medicine and Department of Urology, The First Affiliated Hospital of Nanjing Medical University, Nanjing, China

**Keywords:** Azoospermia, Congenital abnormalities, Ejaculatory duct, Urethra

## Abstract

**Background:**

Retrograde ejaculation can have anatomical, neurogenic, or pharmacological causes. Among these factors, malformation of the prostatic urethra is an uncommon cause.

**Case presentation:**

We describe a 29-year-old Han Chinese man with absence of his verumontanum combined with ejaculatory duct cysts, and no other cause for ejaculatory dysfunction. His verumontanum was replaced by a deep groove adjacent to his bladder neck, which could significantly influence bladder neck contraction. In addition, the large cysts in the ejaculatory duct could obstruct the anterior outlet of his prostatic urethra and prevent seminal fluid flow in an anterograde direction.

There are few reports of retrograde ejaculation associated with congenital malformations of the posterior urethra. Malformations associated with bladder neck laxity and increased tone of the prostatic urethral outlet can contribute to retrograde ejaculation.

**Conclusions:**

Malformation of the prostatic urethra is an uncommon cause of retrograde ejaculation, and can be difficult to treat.

**Electronic supplementary material:**

The online version of this article (doi:10.1186/s13256-016-1150-x) contains supplementary material, which is available to authorized users.

## Background

Retrograde ejaculation (RE), an uncommon cause of male infertility, occurs when semen is directed into the urinary bladder, rather than in an anterograde direction. The patient usually has an orgasm, but little or no semen is ejaculated. Hence, RE is referred to as “dry ejaculation.” RE can be congenital, acquired, or idiopathic; myogenic or neurogenic abnormalities are the primary causes [1]. Many factors have been identified as a cause of congenital RE, including stenosis of the bulbous urethra, defects of the internal urethral sphincter, and congenital bladder neck obstruction [2]. As the causes differ from each other, the treatment of congenital RE can be complex and difficult. Here, we describe an uncommon case of RE combined with malformation of the prostatic urethra.

## Case presentation

**Fig. 1:**
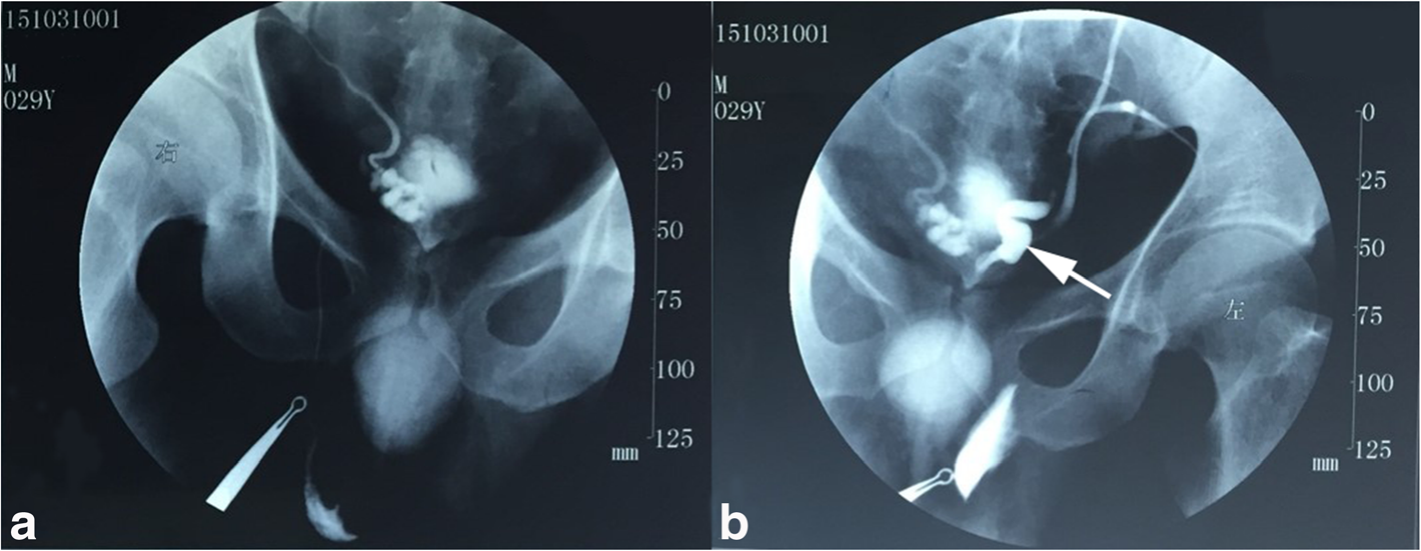
Vaso-seminal vesiculography applied in this patient. **a** The bladder filled with contrast agent injected into the right vas deferens. **b** Contrast agent injected on the left side showed that the end of the left vas deferens was dilated, and the bladder filled with contrast agent. The arrow in panel **b** is pointing to the dilated left vas deferens

**Fig. 2:**
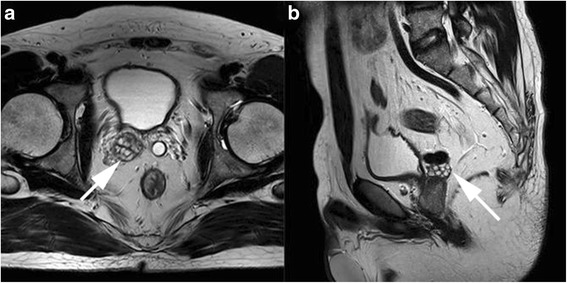
Pelvic magnetic resonance imaging (MRI) revealed left seminal vesicle aplasia combined with right ejaculatory duct cysts. **a** horizontal section. **b** sagittal section. The arrows in **a** and **b** are both pointing to the right ejaculatory duct cysts

A 29-year-old Han Chinese man had a 3-year history of azoospermia. Routine semen analysis demonstrated scant, acidic seminal fluid (1.0 mL) with no spermatozoa; however, sperm cells were found by percutaneous epididymal aspiration. Pelvic magnetic resonance imaging (MRI) and vaso-seminal vesiculography revealed dilation at one end of his left vas deferens and ipsilateral seminal vesicle aplasia; a large cyst was found in the ejaculatory duct adjacent to his prostate on the right (Figs. 1 and 2). His bladder filled with contrast agent during vaso-seminal vesiculography. A postejaculate urine sample showed the presence of semen. Therefore, he was diagnosed as having RE. For further identification of the cause of his RE, seminal vesicle endoscopy was performed. The results demonstrated that the verumontanum was replaced by a deep groove adjacent and inferior to the neck of his urinary bladder. The bilateral ejaculatory duct openings were found in the deep groove and his ducts exhibited cystic dilation, accompanied by dilation of the right seminal vesicle. Moreover, seminal fluid was found in the intra-cavity. No parenchymal obstruction was found in his ejaculatory ducts, seminal vesicles, or vas deferens openings.

RE resulting from a congenital malformation was suspected owing to the following reasons: (1) the contrast agent was visible in the bladder during vaso-seminal vesiculography, (2) the groove that replaced the verumontanum was adjacent to the bladder neck and could affect normal contraction of the bladder neck, (3) cysts in the ejaculatory duct could obstruct the anterior outlet of the prostatic urethra and guide the seminal fluid in a retrograde direction. As a result, we suspected that his RE mainly resulted from the congenital malformation of his prostatic urethra. Moreover, sperm cells were found by percutaneous epididymal aspiration. Because the evaluation was performed to diagnose the cause of infertility, he and his wife were referred to a reproductive clinic for *in vitro* fertilization (IVF) treatment. The treatment was successful, and the couple achieved pregnancy.

## Discussion

There are few reports of RE associated with congenital malformations of the posterior urethra. In male embryos, the paramesonephric duct undergoes degeneration and the mesonephric duct develops into the vas deferens, ejaculatory duct, and seminal vesicle. The urogenital sinus develops into the bladder, prostate, urethra, urethral gland, prostatic urethra, membranous urethra, and penile urethra. In this case, malformation of the posterior urethra may have been associated with the development of the urogenital sinus in the embryonic period. The causes of RE can be anatomical, neurogenic, or pharmacological, and ultimately impair normal anterograde ejaculation. Two distinct phases are involved in the normal physiology of ejaculation: emission and expulsion. Emission, the first stage of ejaculation, refers to deposition of the semen in the posterior urethra through the peristaltic contraction of the epididymis, vas deferens, seminal vesicles, and prostate; expulsion is defined as the transport of the semen through the urethra. Normal expulsion relies on synchronized interplay of many factors, including urinary bladder neck closure, periurethral muscle contraction, and relaxation of the external urinary sphincter. Among these factors, bladder neck contraction plays a vital role in preventing retrograde flow of semen into the bladder, and can reach a pressure of up to 500 cm H_2_O [3]. Neurogenic or myogenic factors interfering with the bladder neck contraction can lead to RE; for instance, injury of the nerves or normal anatomic structure of the bladder neck during transurethral prostatectomy is the most common cause of RE [4]. In addition to closure of the bladder neck, other factors influencing involved expulsion also contribute greatly to anterograde flow of seminal fluid. A urethral stricture, for example, in which stenosis of the bulbous urethra can obstruct the flow of seminal fluid from the posterior urethra, can result in RE. The presence of a posterior urethral valve may also be a cause of anatomical RE [2]. Thus, when there is weakness of bladder neck contraction or a stricture of the urethra, RE is induced by pressure that reduces the flow of seminal fluid in an anterograde direction (bladder neck contraction) and/or pressure preventing anterograde flow of the seminal fluid (urethral stricture).

In this case, malformations of the prostatic urethra, including absence of the verumontanum and the presence of cysts in the ejaculatory duct, could have affected the expulsion process. The verumontanum was replaced by a deep groove adjacent to the bladder neck, which could have affected contraction, thereby decreasing the pressure that causes seminal fluid to flow in an anterograde direction. In addition, cysts in the ejaculatory duct obstructed the anterior outlet of the prostatic urethra, and the increased pressure could have induced RE. Thus, the unusual combination of multiple malformations could have caused RE.

Several approaches have been used in the treatment of RE, including medical therapy (alpha-agonists and tricyclic antidepressants), penile vibratory stimulation, electroejaculation, and bladder neck reconstruction surgery [5]. These methods aim at restoring the normal physiology of anterograde ejaculation. However, congenital RE resulting from an anatomical malformation is rare, and no consensus treatment regimen has been reported. Moreover, most patients with RE seek treatment because of male infertility, and urinary sperm is usually retrieved for use in artificial insemination.

## Conclusions

Malformation of the prostatic urethra is an uncommon cause of RE, and increases the difficulty of treatment. There is no consensus treatment regimen to date.



**Additional file 1**: A video of seminal vesicle endoscopy. (WMV 89.9 MB)


## Abbreviations

IVF: *In vitro* fertilization
MRI: Magnetic resonance imaging
RE: Retrograde ejaculation
